# Special Issue “Advanced Signal Processing in Wearable Sensors for Health Monitoring”

**DOI:** 10.3390/s22062189

**Published:** 2022-03-11

**Authors:** Maysam Abbod, Jiann-Shing Shieh

**Affiliations:** 1 Department of Electronic and Electrical Engineering, Brunel University London, Uxbridge UB8 3PH, UK; 2 Department of Mechanical Engineering, Yuan Ze University, Taoyuan 32003, Taiwan

Wearable sensors are becoming very popular recently due to their ease of use and flexibility in recording data from home. They can range from simple adhesive sensors to more sophisticated, stretchable implants to monitor health or for diagnosis. The basic unit of a wearable sensor is the electrodes or wires, the power source, and the interface/communication unit, which can be a smartphone or other types of signal receivers. One of the most important features of a wearable sensor is flexibility: It has to flex, stretch and twist without straining the sensory part and maintain the quality of the measured signal. Most wearable sensors measure physiological signals and incorporate a real-time decision system to interpret the signal and detect symptoms or measure context awareness. Different system implementations and technological approaches are used for the design of the state-of-the-art in wearable biosensors: some have advantages and some have shortcoming. The literature of such techniques is surveyed in order to provide direction for future research improvements [1].

Technology is continually improving, making many tools and algorithms available to developers with diverse applications and connectivity. Wearable sensors are benefiting from the underlying versatile technologies enabling them to capture rich contextual information that deliver a legitimately personalized experience. The extensive and diverse classification of wearable devices with wireless communication, data processing and on-board classification is reported in a survey that highlights the challenges and future solutions in this field [2].

The market of wearable sensors is growing exponentially, with an annual growth rate of 20%. Moreover, the outbreak of COVID-19 had a tremendous impact on the evolution of wearable device, driven by the requirements of home sensing and diagnosis devices [2]. Many systems have been developed for different applications, such as protection for the elderly, health home monitoring, gait analysis, interactive media and animation that helps people become familiar with such technologies [3].

In this Special Issue, special attention is given to the AI technologies that are utilized in signal processing and diagnosis. Signals usually need filtering, conditioning and processing. Advanced algorithms are computationally intensive and require fast hardware. This can represent a major hurdle in developing such systems, and most developers revert to performing the heavy signal processing on advanced computing systems such as GPUs, computer clusters, cloud applications and edge computing. This serves wearable sensor very well, as information can be transferred via the Internet, providing sophisticated algorithms and high-speed processing power.

Papers published in this Special Issue are focused onto two subjects: health monitoring using biological signal (EEG, ECG) and physical health monitoring (movements). The subject of health monitoring focused on recording vital signs such as brain activities (EEG) and applications to the cardiovascular system (ECG, HR, PPG); this issue includes seven papers in this field. The application of EEG is rather difficult due to the need for good wearable sensors that can detect the signal reliably. However, filtration and signal conditions are some of the techniques that can be used to extract meaningful information from noisy signals. EEG can be used to monitor different activities; one particular example is drowsiness detection, which is very useful for drivers who might lose attention during driving [4]. On the other hand, the use of ECG signal processing using deep learning (DL) algorithms tend to be the latest in the field. One particular application is the monitoring of the hemodynamics using ECG and DL without the need for invasive sensing [5].

Other biological signal applications are mainly related to the cardiovascular system, such as the detection of heart rate and blood pressure using either facial expressions [6] or PPG sensors [7,8] respectively. Such measurements are not easy to realize with high accuracy, hence, there is a need for DL systems to process images or signals in order to obtain good accuracy. Finally, DL is also used for the detection heart rhythm anomalies, and a short survey is presented in [9] that looks at the different techniques utilizing wearable sensors. The final application is the use of ECG for the detection of myocardial infarction (MI), which is one of the most prevalent cardiovascular diseases. An LSTM network is used to detect MI based on ECG signal [10].

Physical health monitoring using wearable sensors is presented in two papers related to physical movements, such as the prediction of joint momentum for the purpose of predicting the force generated by the muscle using an ANN for the purpose of skeleton control [11]. Another related work presented in [12] is based on the detection of chewing event using EMG signals.

The last paper is a mini-review that addresses pain as a subjective feeling. The review presents the correlation between pain and stress, and the measurement approach uses wearable sensors. Various physiological signals (i.e., heart activity, brain activity, muscle activity, electrodermal activity, respiratory, blood volume pulse, skin temperature) as well as expression/behavior are listed as measurable signs using wearables sensors. Wearable sensors used for healthcare monitoring systems can detect pain and stress. As a consequence, pain leads to multiple symptoms such as muscle tension and depression; hence, integrating modern computing techniques with wearable sensor measurements can help in pain control [13].

## Data Availability

Not applicable.
